# Monthly hydropower generation data for Western Canada to support Western-US interconnect power system studies

**DOI:** 10.1038/s41597-025-05098-2

**Published:** 2025-05-28

**Authors:** Youngjun Son, Cameron Bracken, Daniel Broman, Nathalie Voisin

**Affiliations:** 1 Pacific Northwest National Laboratory, Richland, WA USA; 2 Department of Civil and Environmental Engineering, University of Washington, Seattle, WA USA

**Keywords:** Hydroelectricity, Hydrology

## Abstract

Hydroelectric power generation in Western Canada significantly contributes to power grid operations of the North American Western Interconnection through substantial generation, some of which is exported to the United States (U.S.). However, the lack of publicly available hydropower generation datasets poses challenges for future market projections and resource adequacy evaluations. We present a simulation-based monthly power system model-ready hydropower generation dataset for 110 facilities in British Columbia and Alberta from 1981 to 2019. These monthly hydropower generation estimates are developed from integrated hydrologic model simulations of runoff and reservoir-operated streamflow, followed by scaling that considers diversion inflow constraints based on hydropower water license information. To address the lack of comparable hydropower generation records, we conduct step-by-step evaluations for simulated runoff, regulated streamflow, and hydropower generation using available observations or estimates. The presented hydropower dataset aims to enhance the representation of hydropower resources in Western Canada, supporting power grid system studies for the Western Interconnection of the U.S. and Canada.

## Background & Summary

Hydropower serves as a vital, dispatchable energy resource, supporting the integration of other renewable electricity generation across the North American Western Interconnection (WI). In the WI region, which extends from 11 U.S. states to British Columbia and Alberta in Canada, as well as to northern Baja California in Mexico, hydroelectric power generation accounted for between 24% and 30% of the annual electricity production from 2012 to 2022, amounting to 219.2 *TWh* in 2022^1,2^. Notably, in British Columbia, about 90.9% (equivalent to 62.6 *TWh*) of the provincial electricity generation originates from hydropower in 2022^3^. British Columbia also has substantial electricity imports and exports with the U.S. to maintain power grid system reliability^4^. This electricity trade is a key component in ensuring grid reliability within the WI when evaluated by the Western Electricity Coordinating Council (WECC), the organization responsible for the reliability of the bulk power system.

Coincident hydropower generation datasets across the WI region of the U.S. and Canada are essential for resource adequacy assessments and bulk power grid reliability studies. Particularly, the growing load demands driven by ongoing electrification^2^ necessitate unprecedented regional coordination on the use of technologies with storage capabilities, like hydropower. However, there is currently a gap in the availability of consistent transboundary hydropower generation datasets, primarily due to differences in governance policies in the two countries. In the U.S., for example, the Energy Information Administration (EIA) routinely collects electricity generation data from power plant operators through monthly and annual surveys (EIA-923, formerly EIA-906/920, https://www.eia.gov/electricity/data/eia923/), which are openly accessible. Moreover, measured timeseries datasets of hydroelectric power generation are readily available for facilities operated by U.S. federal agencies, such as the U.S. Army Corps of Engineers (Corps Water Management System, https://cwms-data.usace.army.mil/cwms-data/) and U.S. Bureau of Reclamation (Reclamation Information Sharing Environment, https://data.usbr.gov/rise-map/). These accessible datasets have facilitated subsequent research^5^, including extended datasets^6^ and future energy projections^7,8^, to support power system modeling and simulations for electricity market projections and resource adequacy evaluations. Conversely, in Western Canada, publicly available hydropower generation datasets are sparse and vary in their representation and level of aggregation, as summarized in Table 1. For instance, Statistics Canada, a government agency of Canada, provides monthly provincial- and territorial-level totals of hydropower generation (Canadian Centre for Energy Information, https://energy-information.canada.ca/en). Additionally, other generation estimates are only indirectly available for specific years, such as monthly profiles for 2015^9,10^ and annual totals from 2013 to 2017^11^, none specifically vetted by the Canadian government or utilities. Consequently, the data availability gap poses challenges for integrated WI-wide power system studies, including inconsistency in data scales and imbalance in data representation, which lead to uncertainties in regional grid reliability studies and associated locational marginal prices to inform regional capital investments^5^. Furthermore, these challenges become particularly prominent in regions such as the Pacific Northwest, where hydropower generation is impacted by changes in climate, glacial mass, snowpack levels, and sediment loads^12–21^.

**Table 1:** Publicly available hydropower generation datasets for facilities in Western Canada.

Dataset	Type	Spatial representation	Temporal representation	Modeling Approach		
				Runoff	Streamflow and reservoir operations	Hydropower
Statistics Canada^3^ (Canadian Centre for Energy Information)	Survey records	Aggregated by province, territory, or producer class	Monthly since 2008			
WECC ADS 2032^34^ (Last updated for Canada in ADS 2026)	Survey records (planning)	Generator-level (Western Interconnection)	Monthly for 2008 only			
PLEXOS-World 2015^9,10^	Streamflow-based estimates	Utility-level (145 facilities)	Monthly for 2015 only	LPJmL model simulations at 0. 5° × 0.5°^72,73^	Streamflow downscaling at 15″ × 15″^62^ (no reservoir operations)	Calibration (scaling) with a power system model against country-level generation data (IRENA)^70^, with 4^th^ highest month streamflow constraints
World Resources Institute, Global Database of Power Plants^11^	Statistical estimates (machine learning)	Utility-level (145 facilities)	Annual from 2013 to 2017	ERA5 runoff^38^ accumulations over drainage areas	ERA5 streamorder^38^ only	Gradient Boosted Regression Trees, trained by nameplate capacity, average capacity factor by region (EIA) or by country (IRENA)^70^, and ERA5 data^38^
Presented Dataset^66^	Regulated streamflow-based estimates	Utility-level (110 facilities)	Monthly from 1981 to 2019	VIC model simulations at 1/16° × 1/16°	mosartwmpy model simulations with generic reservoir operation rules at 1/8° × 1/8°	Scaling with WECC ADS 2032^34^, integrating diversion inflow constraints

To address the gap in data availability, we develop a dataset of monthly generation estimates for hydropower facilities in Western Canada to support power grid system studies within the WI region. The developed dataset covers 110 facilities located in British Columbia and Alberta, spanning the historical years from 1981 to 2019. The modeling approach for the WI region in Canada involves hydrologic modeling of runoff and streamflow, as similarly applied in the contiguous United States (CONUS)^22,23^. However, due to the limited availability of observed data, the translation into a hydropower generation dataset differs from the CONUS approach, and we use a subsequent hydropower scaling based on simulated streamflow outputs.

For the historical hydrologic simulations based on the meteorological forcing of the perturbed thermodynamics simulations^24,25^, we implement the Variable Infiltration Capacity (VIC) model^26,27^, incorporating grid-based automatic calibration for runoff through an optimization tool^28^. The generated surface and subsurface runoff are routed with reservoir operations using the Model for Scale Adaptive River Transport with Water Management (mosartwmpy)^29–31^. The simulated runoff and regulated streamflow from these hydrologic models are evaluated against a target runoff dataset^32,33^ for calibration and gauge observations (Environment Canada: Water Level and Flow, https://wateroffice.ec.gc.ca/) near hydropower plants, respectively. The evaluations indicate satisfactory performance in mountainous regions where hydropower plants are mostly installed, demonstrating reasonable representations of hydrologic processes and reservoir operations.

We leverage the WECC Anchor Data Set (ADS) 2032^34^, which contains the most recent power plant database to date. The reported monthly hydropower generation in Canada, however, dates from the ADS 2026 case and represents electricity generation for the 2008 weather year while that in the CONUS was updated for the ADS 2032 case using the 2018 weather year. Without further observed generation from hydropower facilities in Western Canada, we scale those reported generation based on monthly regulated flow deviations from this reference 2008 weather year. For the streamflow-hydropower scaling, we consider diversion intake flow rates obtained from hydropower water license information (British Columbia: Water License Viewer, https://j200.gov.bc.ca/pub/ams/; Alberta: Authorization Viewer, https://avw.alberta.ca/) as additional constraints. The hydropower generation estimates are compared with observations^3^ and other available estimates^9–11^ at different levels of aggregation. The comparisons indicate consistency with observed data and improvements over other estimates, especially in aligning with seasonal variations in hydropower generation.

Monthly generation constraints for power grid system studies include additional generation estimates, such as minimum and maximum hourly generation and daily fluctuations. These additional estimates are calculated by applying parameters^35^ derived from hourly hydropower generation records in the U.S. Pacific Northwest within the WI region (U.S. Army Corps of Engineers Northwestern Division, https://www.nwd-wc.usace.army.mil/dd/common/dataquery/www/). Therefore, the developed dataset can contribute to enhancing the representation of coincident hydropower resources within the WI region, as part of the WECC ADS 2034. Presently, resource adequacy studies are performed independently in Canadian provinces using proprietary data. While the presented dataset is currently limited for such studies until hydrologic simulations cover the entire provincial regions and cold climate river processes (e.g., river ice), it can support resource adequacy studies across Canadian provinces and U.S. states.

## Methods

### Overview

**Fig. 1:**
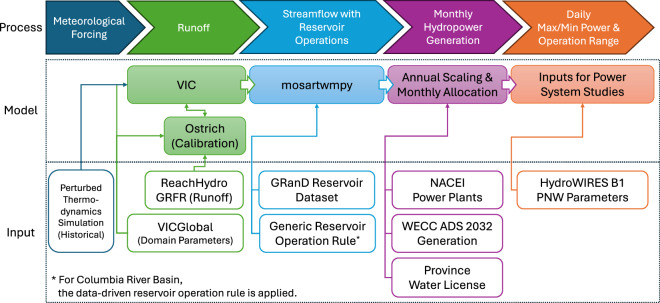
Schematic flow diagram illustrating the processes, models, and inputs to estimate hydropower generation through an integrated hydrology-hydropower modeling approach. Refer to the corresponding subsections for citations.

The monthly estimates of hydroelectric power generation by individual plants in Western Canada are developed using an integrated hydrology-hydropower modeling approach (Type 3a, according to the classifications of large-scale hydropower studies^36^). This integrated approach involves simulating hydrologic processes, including reservoir operations, and subsequently scaling the simulated outputs to estimate hydropower generation. For hydrologic simulations, we extend a modeling approach similar to that presented for the CONUS-wide hydropower estimates^22,23^ into the watersheds of British Columbia and Alberta in Canada. This extension ensures consistent hydrologic representations of river drainage basins spanning the Western U.S. and Canada, serving as a robust basis for hydropower modeling. As outlined in Fig. 1, a hydrologic model, VIC^26,27^, is implemented to generate surface and subsurface runoff based on calibrated, grid-based hydrologic parameters. This runoff is subsequently routed overland through streamflow networks and regulated reservoir storages using a river-routing model, mosartwmpy^31^. To estimate hydroelectric power generations, we apply a streamflow-hydropower scaling approach, which differs from the statistical approach used for the CONUS-wide hydropower estimates^22,23^ due to the limited availability of utility-level reference generation datasets in Western Canada. Each modeling step is described in detail within the following subsections.

### Hydroelectric power plants

**Fig. 2:**
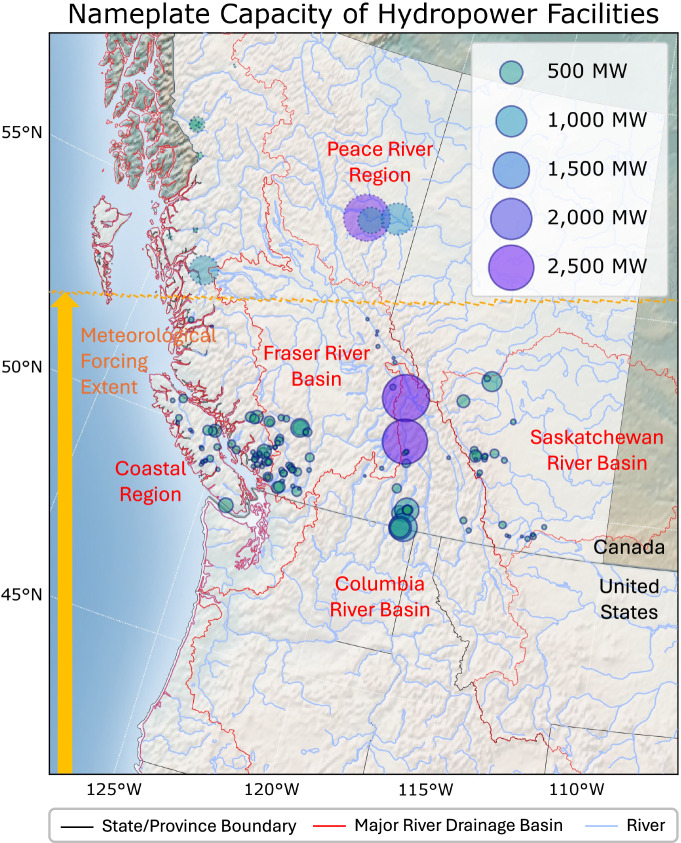
Hydroelectric power plants and associated river drainage basins within the WI region of Canada. The orange arrow shows the spatial coverage of the applied meteorological forcings, with the northern boundary delineated by the dashed orange line. The hydropower facilities outside the forcing extent are shown as light circles with dotted outlines.

According to the North American Cooperation on Energy Information (NACEI)^37^, 146 hydropower plants with generation capacities greater than 1 *MW* are initially identified in British Columbia (123 facilities) and Alberta (23 facilities). Figure 2 illustrates their locations and nameplate capacities, in addition to their associated river drainage basins. Several facilities with nameplate capacities exceeding 1,000 *MW* are located in the Columbia River Basin in southern British Columbia and the Peace River region in the northern part of the province, which are characterized by extensive reservoir storage with regulation. Other small- to mid-capacity facilities are distributed across the mountain ranges within the Fraser River Basin, the Saskatchewan River Basin, and coastal regions, particularly along the Canadian Cascade and Canadian Rocky Mountains. These facilities predominantly belong to the run-of-river category, where water is diverted from diversion intakes to powerhouses through long penstocks, with little or small storage involved.

Out of the 146 identified hydropower plants, 36 facilities are excluded from the dataset generation process due to the limited spatial coverage of the applied meteorological forcings over northern Canada (15 facilities, indicated as light circles with dotted outlines in Fig. 2) and the lack of corresponding facilities in the reference generation dataset, WECC ADS 2032^34^ (21 facilities). Notably, in northern British Columbia, the facilities located within the Peace River region (Gordon M. Shrum, Peace Canyon, and Site C) and the inter-basin transfer facility (Nechako-Kemano) account for most of the excluded capacities, approximately 5,400 *MW*. However, the hydropower generation at Nechako-Kemano (896 *MW*) is mainly consumed by a nearby smelter. In addition, among the 21 facilities (261.4 *MW* in capacity) which generation is not identified in WECC ADS 2032, there are those primarily built to supply power to local industries (e.g., mine mills at Tennant Lake with 3.1 *MW* and at Thelwood with 8.2 *MW*, a pulp mill at Lois Lake with 37 *MW*, and an LNG export terminal at Squamish Power Project with 1 *MW*) and those that have halted operations (e.g., Lake Buntzen 2 with 17 *MW*). Since the generation from these hydropower plants is not primarily used for transmission, their impacts are likely limited for power grid system studies across the WI region. Nevertheless, a few large facilities, such as Forrest Kerr (195 *MW*), Montrose (73 *MW*), and those within the Peace River region, need to be further integrated in future studies to make the dataset more comprehensive by leveraging available generation records or by expanding meteorological forcings. In our study, although hydropower plants may have been newly installed, upgraded, or decommissioned between 1981 and 2019, it is assumed that their power generation characteristics have remained constant. Table 2 summarizes the statistics of the hydroelectric power plants that are considered in our dataset.

**Table 2:** Summary statistics of hydroelectric power plants considered in our dataset.

Province	Facilities	Capacities	Excluded facilities and capacities
British Columbia	123	16,971.5 *MW*	No coverage: 15 (5,775.3 *MW*) No reference generation: 15 (210.9 *MW*)
Alberta	23	934.2 *MW*	No reference generation: 6 (50.5 *MW*)
Total	146	17,905.7 *MW*	36 (6,036.7 *MW*)

### Meteorological forcing input

The 40-year historical run (1980–2019) of the perturbed thermodynamics simulations^24,25^ is applied as meteorological forcings in a hydrological model. The climate simulations are based on the dynamical downscaling of the European Centre for Medium-Range Weather Forecasts Version 5 Reanalysis (ERA5)^38^ using the Weather Research and Forecasting (WRF) model^39^. The datasets feature a spatial resolution of approximately 12 *km* over the CONUS, including southern Canada and northern Mexico, and an hourly temporal resolution for surface atmospheric variables. Because of their spatial extent, which covers up to the dashed line in Fig. 2, we exclude the northern portions of Canada, including watersheds with hydropower facilities. Most of the excluded portions belong to separate river drainage basins, such as the Pacific Coast and the Mackenzie River (Peace River). The excluded headwater portions within the Fraser River Basin flow into its mainstem, where no hydropower facilities are installed. While these limitations restrict provincial resource adequacy studies, the presented dataset remains responsive to studies that span multi-provinces and the WI region. Nevertheless, the climate simulations are employed with an emphasis on maintaining consistency with the CONUS-wide hydropower estimates^22,23^ and potential extensions for future projections based on the perturbed thermodynamics scenarios. Among the numerous atmospheric variables, precipitation, temperature, surface pressure, both long-wave and short-wave fluxes, wind speed, and vapor pressure are extracted with 6-hour intervals and bi-linearly interpolated from about 12 *km* (1/8^th^ degree) WRF grids into 1/16^th^ degree grids as meteorological forcing inputs for generating runoff in a hydrological model.

### Runoff generation

The hydrologic modeling procedure for generating runoff is identical to that presented for the CONUS-wide hydropower estimates^22,23^. The VIC model^26,27^ is implemented to generate 6-hourly surface and subsurface runoff through hydrologic processes. It solves water and energy balances at each grid cell to calculate hydrologic states and fluxes on land surfaces. Prior to calibration, the hydrologic parameters of the land-surface domain are initialized using the VICGlobal dataset^40,41^ that has a resolution of 1/16^th^ degree. The VICGlobal dataset consists of soil parameters derived from the soil texture classifications of the Food and Agriculture Organization Harmonized World Soil Database^42^ and vegetation parameters derived from Moderate Resolution Imaging Spectroradiometer (MODIS) remote sensing measurements. The detailed methodology for deriving the hydrologic parameters can be found in the data descriptor^40^.

To calibrate a subset of the hydrologic parameters, the simulation period is divided into two phases: a calibration period from 1981–2000 following a spin-up year of 1980 and a validation period from 2001–2019. As listed in Table 3, the calibration parameters are selected from commonly adjusted VIC parameters found in existing literature^32,40,43,44^. The Optimization Software Toolkit for Research Involving Computational Heuristics (OSTRICH) model^28^ is integrated for automatic calibration, where the Dynamically Dimensioned Search (DDS) algorithm^45^ is applied to the selected calibration parameters for each 1/16^th^ degree grid. The objective function is defined to maximize the modified Kling-Gupta Efficiency (KGE) metric^46^ for total monthly runoff, in comparison to the Global Reach-level Flood Reanalysis (GRFR) ReachHydro daily runoff dataset^32,33^, as written in Eq. (1):

1 $${\rm{KGE}}{\prime} =1-\sqrt{{\left(r-1\right)}^{2}+{\left(\beta -1\right)}^{2}+{\left(\gamma -1\right)}^{2}}$$

where *r* is the correlation coefficient between simulated and observed runoff, *β* represents the bias ratio of the simulated mean to the observed mean, and *γ* denotes the variability ratio of the simulated coefficient of variation to the observed coefficient of variation.

**Table 3:** Selected VIC parameters and their ranges for calibration.

Parameter	Description	Unit	Min	Max
*b*	Shape Parameter for Variable Infiltration Capacity Curve	—	0.001	0.8
*D*_*m*_	Maximum Baseflow Velocity	*mm/day*	1	30
*D*_*s*_	Fraction of *D*_*m*_ for Linear Baseflow Curve	Fraction	0	1
*W*_*s*_	Fraction of Maximum Soil Moisture for Linear Baseflow Curve	Fraction	0.5	1
*d*_*2*_	Thickness of Intermediate Soil Layer	*m*	0.1	2
*d*_*3*_	Thickness of Bottom Soil Layer	*m*	0.1	2
*Expt*_*2*_	Brooks-Corey Exponent for Intermediate Soil Layer	—	8	30
*Expt*_*3*_	Brooks-Corey Exponent for Bottom Soil Layer	—	8	30

### Streamflow routing with reservoir operations

The surface and subsurface runoff generated by the calibrated VIC model are aggregated daily to a 1/8^th^ degree resolution with an area-weighted approach and subsequently routed using the mosartwmpy model^31^, a Python translation of the Model for Scale Adaptive River Transport with Water Management (MOSART-WM)^29,30^. The MOSART-WM model consists of two primary components: a kinematic-wave flow routing solver and a water management solver. The flow routing solver facilitates the transport of daily runoff through hillslopes, tributaries within each grid, and main channels along connected grids, regulated by reservoir operation rules defined for irrigation, water supply, flood control, and hydropower generation at designated reservoir storage grids. The model hydraulic parameters are obtained from global model applications^47,48^, while reservoir locations, functions, and characteristics are extracted from the Global Reservoir and Dams (GRanD) v1.3 dataset^49^.

For reservoir operations, generic operation rules^30,50,51^ are chosen for their simplicity, given the lack of readily accessible reservoir release and water demand datasets in Western Canada. Within the generic operation rules, the target releases are determined by the primary reservoir functions, such as irrigation and non-irrigation purposes, and the flood control priority. The target releases are subsequently adjusted to account for interannual variability of inflows, seasonal variability in water demand, and monthly release and storage targets to accommodate conflicting objectives like irrigation and hydropower. Minimum environmental flow and storage levels also inform daily release decisions. The rule details are described in the literature^30,50,51^. The generic operation rules for reservoirs have been effectively implemented to represent water management components, enabling an understanding of water availability and its implications on hydropower generation across the WI^14–16,18,52,53^, CONUS^54–56^, and southern Canada^57^ regions. Currently, water withdrawal and supply allocation processes are not included in the representation of water management in Western Canada due to the lack of distributed water demand datasets, which should be integrated in future studies for further enhancement. Several Canadian hydropower plants are located within the Columbia River Basin that spans the U.S.-Canada border (see Fig. 2). Consequently, their monthly generation is influenced by hydrologic processes occurring in the U.S. portion of the basin. To utilize the enhanced representation of reservoir operations in the U.S. and maintain watershed consistency across the national boundary, the streamflow outputs for the Columbia River Basin are directly taken from the CONUS-wide modeling results^22,23^, where more sophisticated, data-driven operation rules^58^ have been developed using measured reservoir release and water demand datasets. However, the generic operation rules are still applied to other river drainage basins in Western Canada.

The accurate selection of grid locations, which are relevant to hydropower generation, is essential for extracting simulated streamflow outputs for hydroelectric power plants. In Western Canada, however, many run-of-river facilities have diversion intakes installed at a distance from their powerhouses to gain hydraulic heads for generation. In addition, the river networks represented by the 1/8^th^ degree grids may not accurately reflect open channel connections, including confluence or divergence points, around the water sources^8^. To address these spatial gaps, the representative grid locations of the diversion intakes are determined based on provincial hydropower water license information in British Columbia (Water License Viewer, https://j200.gov.bc.ca/pub/ams/) and Alberta (Authorization Viewer, https://avw.alberta.ca/), and then adjusted to align with the river networks delineated for the mosartwmpy model. The specific links to the water license information for individual hydropower plants are available within Hydropower Facility List described in the *Data Records* section.

### Hydropower annual scaling and monthly allocation

**Fig. 3:**
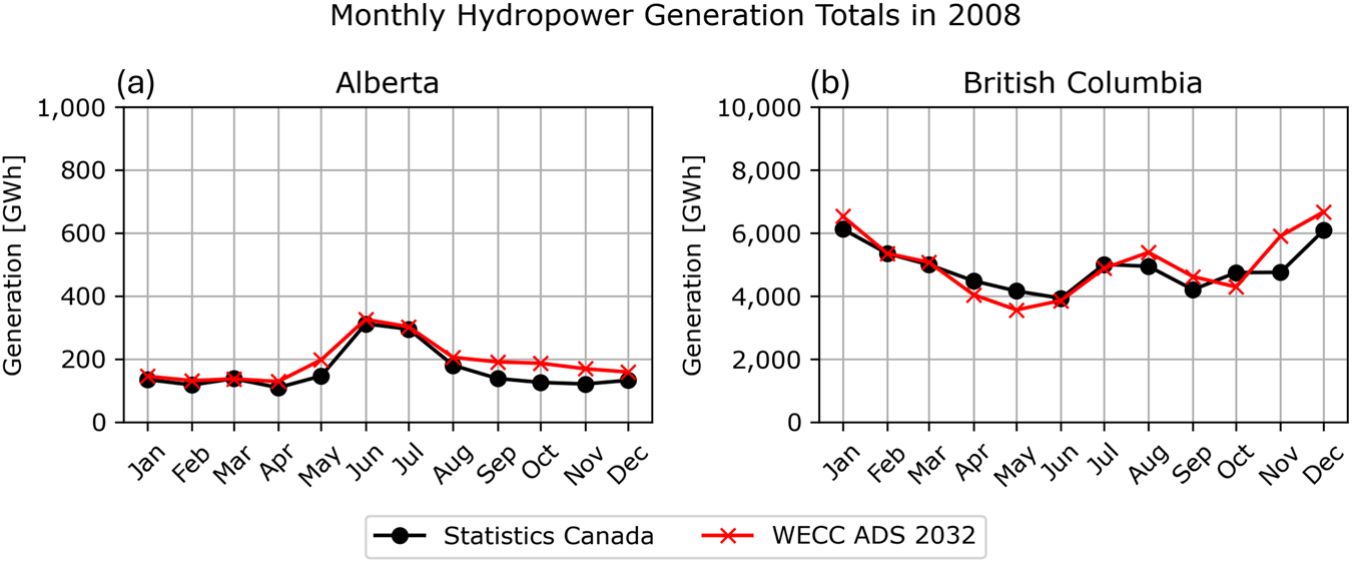
Comparison of WECC ADS 2032^34^ with Statistics Canada^3^: (**a**) Alberta and (**b**) British Columbia. The WECC ADS 2032 plots (red) show the aggregated monthly generation from hydropower facilities listed in Table 2, while the Statistics Canada plots (black) indicate the reported provincial monthly generation statistics.

The daily simulated streamflow outputs are aggregated and scaled to estimate monthly hydropower generation, leveraging WECC ADS 2032^34^. The reference generation dataset contains the baseline power grid system conditions for future reliability studies, including generator-level monthly hydropower generation data reported for the year of 2008 in British Columbia and Alberta for Western Canada. Figure 3 shows that the aggregated monthly hydropower generation from WECC ADS 2032 aligns closely with the actual provincial statistics reported by Statistics Canada^3^. For the 110 hydropower plants listed in Table 2, the dataset provides monthly generation for generators at facilities with large reservoir storages in the Columbia River Basin, namely Mica, Revelstoke, and Arrow Lake, and annual total generation for generators at other facilities. The generator-level generation is aggregated at individual hydropower facilities to obtain the utility-level generation. Since WECC ADS 2032 primarily aims at planning with single-year coverage, it may not be representative of hydropower generation throughout all historical periods. Nonetheless, WECC ADS 2032 is utilized to develop our dataset because it is the only publicly accessible generation records at the generator level for facilities located in Western Canada (see Table 1).

Annual hydropower generation shows strong correlations with annual streamflow, particularly in the Pacific Northwest region^59,60^. Based on these correlations, the conversion of streamflow, or even runoff, into generation is often modeled by introducing linear scaling factors^21,61^, which are subsequently disaggregated using monthly generation patterns^14–17,53^. For regulated streamflow outputs, our study applies a similar scaling approach using WECC ADS 2032. Provided with sufficient generation records and corresponding streamflow data, the scaling approach can be refined into a statistical regression approach^18,58^ based on multi-year conditions that accurately reflect their interannual variabilities. The streamflow-hydropower scaling process involves two main steps: annual total scaling and monthly allocation. First, annual total scaling derives scaling factors for hydropower generation relative to streamflow volumes on an annual basis for individual facilities and applies them to annual streamflow volumes in other years, as written in Eq. (2). Then, monthly allocation disaggregates the annual hydropower generation into monthly intervals based on the monthly distributions of streamflow volumes within each year, as shown in Eq. (3).

2 $${\rm{Annual}}\,{\rm{Total}}\,{\rm{Scaling}}=\frac{{\rm{Total}}\,{\rm{Hydropower}}\,{\rm{Generation}}\,{\rm{in}}\,2008}{{\rm{Total}}\,{\rm{Streamflow}}\,{\rm{Volume}}\,{\rm{in}}\,2008}\times {\rm{Annual}}\,{\rm{Streamflow}}\,{\rm{Volume}}$$

3 $$\text{Monthly Allocation}\,=\,\text{Annual Total Scaling}\times \frac{\text{Monthly Streamflow Volume}}{\text{Annual Streamflow Volume}\,}$$

Streamflow typically peaks during the summer, due to substantial snowmelt. According to Eq. (3), large streamflow peaks concentrated over specific months can lead to excessive estimates of hydropower generation, compared to the facility’s nameplate capacity. Therefore, monthly estimates are adjusted not to exceed the maximum possible generation based on the nameplate capacity. To account for turbine design capacity and possible water spills, additional capping on daily streamflow can be applied to reflect flow constraints, including those based on flow statistics (e.g., 90^th^ percentile of long-term mean streamflow^6^ or 4^th^ highest monthly streamflow^9,62^). In our dataset, diversion intake flow rates, which are collected from hydropower water license information in British Columbia (Water License Viewer, https://j200.gov.bc.ca/pub/ams) and Alberta (Authorization Viewer, https://avw.alberta.ca/), serves as a daily maximum cap when calculating streamflow volumes in Eqs. (2) and (3). The provincial hydropower water license information is available for all 93 hydropower plants in British Columbia and for 10 out of 17 facilities in Alberta. For those facilities in Alberta that lack intake flow rate information (approximately 1.2% of the total nameplate capacity in British Columbia and Alberta), only their nameplate capacities are used to cap the monthly estimates of hydropower generation.

**Fig. 4:**
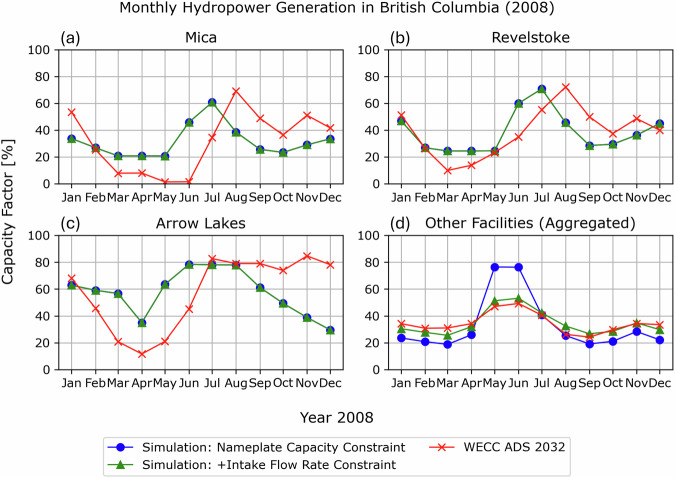
Comparisons of monthly capacity factor estimates with WECC ADS 2032^34^ for (**a**) Mica, (**b**) Revelstoke, (**c**) Arrow Lakes, and (**d**) aggregated other facilities. The capacity factors are calculated using the nameplate capacities of the facilities. The blue and green lines represent the simulated estimates constrained by nameplate capacities and by additional intake flow rates, respectively. Both lines overlap for the (**a**) Mica, (**b**) Revelstoke, and (**c**) Arrow Lakes facilities, which are associated with large reservoir storages. The red lines indicate WECC ADS 2032, which annual totals serve as a basis for hydropower scaling.

Figure 4 shows a comparison of the monthly capacity factor estimates, integrating an additional cap of intake flow rates on daily streamflow volumes (green line). Compared to those with the monthly capping by the facility’s nameplate capacity (blue line), the additional constraint has little impact on the hydropower generation estimates for facilities with large reservoir storages (Fig. 4a–c; Mica, Revelstoke, and Arrow Lakes), while the aggregated monthly totals for other facilities (Fig. 4d) closely approximate WECC ADS 2032 (red line). As these other facilities include many run-of-river facilities with small storage capacity, their monthly generation peaks during the high flow seasons^6^, necessitating reasonable streamflow capping to prevent overestimated scaling. On the other hand, facilities with large reservoir storages show little sensitivity (no difference between blue and green lines) to the additional constraint because their intake capacities are accordingly large. The WECC ADS 2032 capacity factors for the three hydropower plants indicate seasonal patterns that lag by a few months from the high flow seasons, with relatively low generation during spring to summer and winter-peaking in fall to winter. These noticeable lags in generation are not adequately reproduced by the hydropower scaling based on the streamflow simulations using the generic reservoir operation rules, likely due to the limited representation of water management practices for reservoir regulation. Large hydropower facilities can significantly alter streamflow seasonality through water retention, regulation, and diversion^13^, requiring more data on reservoir operations to develop sophisticated model rules. With the lack of such details, the annual generation totals for the Mica, Revelstoke, and Arrow Lakes facilities are updated for each year, but their monthly allocations are directly extracted from the WECC ADS 2032 generation for 2008 and applied identically to other years. In future studies, the presented dataset could be further improved by integrating accurate water management practices and hydropower generation datasets into refined reservoir operation simulations and subsequent hydropower estimations, respectively. For instance, data mining techniques^5^ can be implemented to systematically collect near real-time operational observation or forecast data (typically available for the most recent few days) and planning documents. Based on the collected data, more advanced approaches, such as data-driven methods^58,63^, can be applied to derive water management practices and inform reservoir operation simulations.

For WI-wide power system studies, the inputs for Production Cost Models (PCMs) and Capacity Expansion Models (CEMs) are additionally produced for 110 hydropower plants based on the monthly generation estimates for 1981–2019. The input variables include monthly generation constraints of maximum, minimum, and daily operation range. Given the lack of observed generation datasets at finer time scales to define these variables for individual facilities in Western Canada, we leverage empirical parameters relative to monthly mean generation by facility type (either reservoir storage or run-of-river)^35^. The empirical parameters are derived from the EIA hydropower generation records (https://www.eia.gov/electricity/data/eia923/) and their imputations^6^ for 2001 to 2022 in the U.S. Pacific Northwest within the WI region.

The estimated power system model-ready hydropower dataset from 1981 to 2019 includes monthly potential hydropower generation and associated flexibility metrics (minimum and maximum hourly generation and daily fluctuations). Our dataset covers the WI region in Western Canada and supports power system studies over the entire WI, enabling spatial and temporal coincidence in hydropower representation along with the CONUS-wide hydropower estimates^22,23^. While this dataset cannot be used to design water-energy operations under extreme events or support contingency analyses, it can be used for informing large-scale long-term electricity planning. Specifically, the presented dataset focuses on the importance of coincidence and inter-annual variability, which are key to inform regional dependencies and price signals. This dataset can also be utilized for evaluating future infrastructure needs, such as long-term energy storage and transmission across regions^15^, hence assessing proposed investment policies. However, the dataset is not intended to inform cross-border cooperation, which would require higher fidelity models; it can help motivate the need to evaluate specific events to be simulated with higher fidelity models. Furthermore, the data availability of multiple water year conditions will support the update and enhancement of previous studies that were focused solely on the U.S.^8,18,21,56,61,64,65^.

## Data Records

The dataset is available at the following Zenodo repository^66^: 10.5281/zenodo.14984725.

The presented dataset contains the following simulation-based monthly hydropower generation data for 110 facilities in British Columbia and Alberta, to support Western-US interconnect grid system studies:

1. Monthly hydropower generation estimates
2. Monthly hydropower flexibility metrics (minimum and maximum hourly generation and daily fluctuations)

The hydropower generation estimates are provided with reference to the facility list that contains the corresponding metadata for each facility.

### Hydropower facility list

The file, *CAN_hydropower_facilities.csv*, provides essential information on 146 hydropower facilities in British Columbia and Alberta, derived from NACEI Renewable Energy Power Plants, 1 *MW* or more, by Energy Source^37^. Additionally, the facility information has been updated with corresponding National Hydrographic Network (NHN) Work Units^67^, global reservoir and lake database (GRanD^49^ and HydroLAKES^68^), diversion intake flow rates based on water license information, and so on. Below are the descriptions for each column in the facility metadata:

- *fid*: Facility id according to NACEI data. New four-digit id starting with ‘9’ are assigned for facilities with no fid in NACEI data
- *Facility*: Name of the facility
- *X*: Longitude of the facility’s powerhouse
- *Y*: Latitude of the facility’s powerhouse
- *Province*: Province where the facility is located
- *Hydro_MW*: Nameplate capacity of the facility
- *NHN_Work_U*: Associated NHN Work Units
- *GRanD_ID*: Associated reservoir id from the GRanD dataset
- *HydroLAKES_ID*: Associated lake id from the HydroLAKES dataset
- *GINDEX*: Grid id from the mosartwmpy Canada model
- *GINDEX_CONUS*: Grid id from the mosartwmpy CONUS model, used for facilities in the Columbia River Basin
- *Basin_Note*: Indicator for facilities located in the Columbia River Basin or outside of the meteorological forcing domain of the perturbed thermodynamics simulations
- *WECC_ADS_2032*: Indicator for facilities without the WECC ADS 2032 reference hydropower generation data
- *Intake_Flow_Rate*: Diversion intake flow rates based on hydropower water license information Type: Type of facility
- *Water_License*: Link to the source of water license information
- *Scaling*: Annual total scaling factor (total hydropower generation / total streamflow volume for 2008)
- *Scaling_IntakeCap*: Annual total scaling factor, constrained by intake flow rates from hydropower water license, (total hydropower generation / total streamflow volume not exceeding intake flow rate constraint for 2008)

Among the 146 hydropower facilities listed, only 110 facilities, which are within the applied meteorological forcings domain and have reference hydropower generation data, are considered for monthly hydropower generation estimates.

### Monthly hydropower generation estimates and flexibility metrics

Each file contains a monthly timeseries dataset (rows: monthly timestamps, Year-Month) from 1981 to 2019 for 110 facilities (columns: *Facility* listed in **CAN_hydropower_facilities.csv**).

1. **CAN_hydropower_monthly_generation_MWh.csv**: monthly total hydropower generation in *MWh*
2. **CAN_hydropower_monthly_p_min_MW.csv**: monthly flexibility metric of minimum generation capacity in *MW*
3. **CAN_hydropower_monthly_p_max_MW.csv**: monthly flexibility metric of maximum generation capacity in *MW*
4. **CAN_hydropower_monthly_p_ador_MW.csv**: monthly flexibility metric of the daily operation range in *MW*

Detailed records of the cited datasets can be found in the indicated references within the *Method* section. Monthly hydropower generation data in the WECC ADS PCM cases^34^ are considered public and can be requested to the WECC.

## Technical Validation

### Generated total runoff

**Fig. 5:**
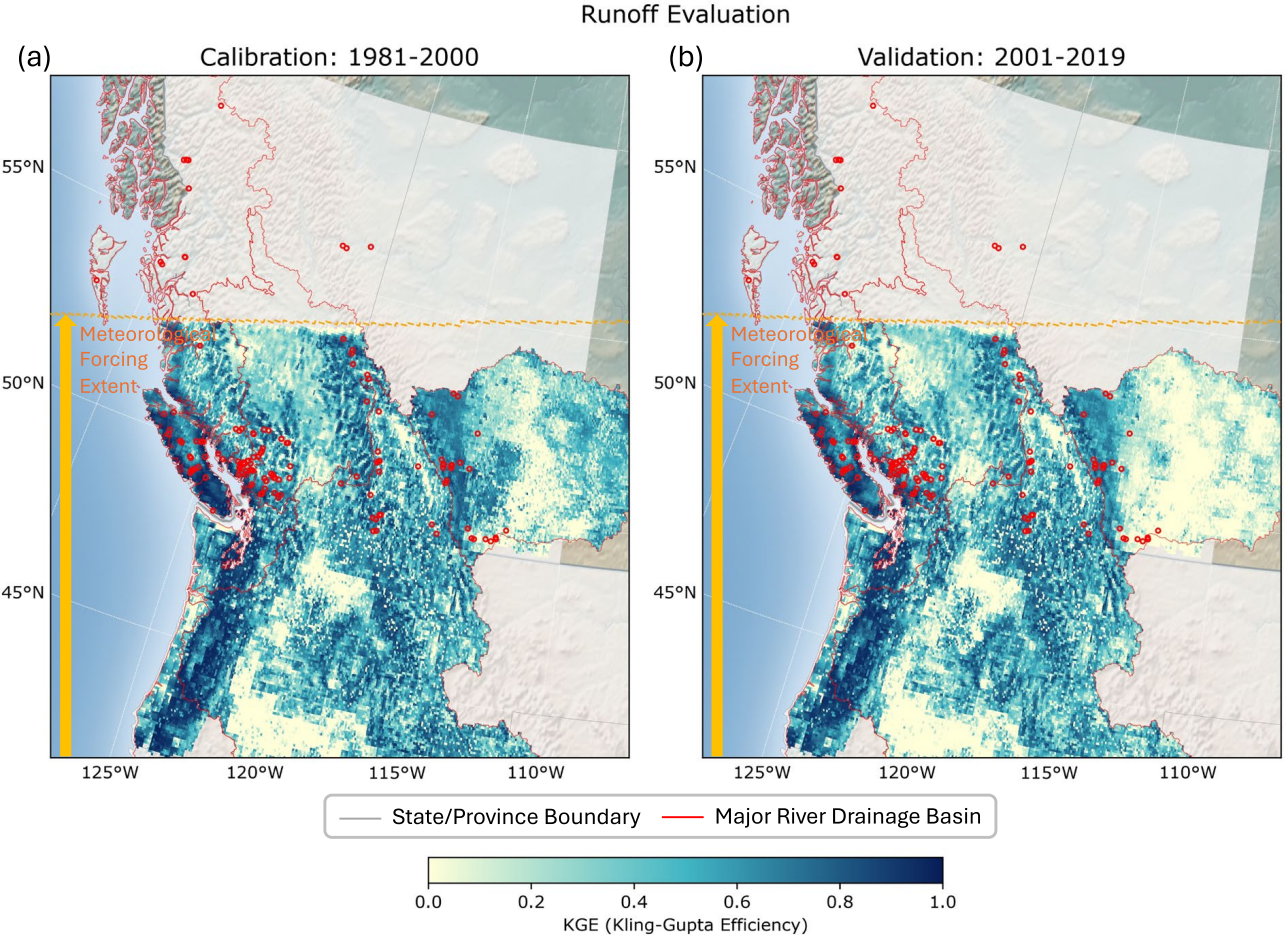
Evaluations of simulated runoff totals with GRFR Reach Hydro daily runoff^32,33^: (**a**) calibration years (1981–2000) and (**b**) validation years (2001–2019). The red dots indicate the locations of hydropower plants as illustrated in Fig. 2. The northern portion of the Fraser River Basin is missing due to the spatial extent of the applied meteorological forcings, covering up to the dashed orange line.

The simulated total runoff from the VIC model is evaluated against the GRFR ReachHydro daily runoff dataset^32,33^ used for calibration. Figure 5 shows the modified KGE values computed from Eq. (1) for the calibration period (1981–2000; Fig. 5a) and the validation period (2001–2019; Fig. 5b), respectively. The same metric values for the Columbia River Basin are incorporated from the CONUS-wide modeling results^22,23^ to maintain hydrologic consistency across the national and watershed boundaries. In Western Canada, the simulated total runoff shows generally reasonable performance (KGE > 0.5) with negligible differences between the two evaluation periods. Mountainous watersheds, where most hydropower plants are located, show relatively high KGE values, while flat regions indicate poor evaluation metrics (KGE < 0.2), particularly in southeastern Alberta. These regions are well-known for prairie pothole features where substantial runoff can be trapped in numerous depression wetlands with complex hydrologic interactions^69^.

As described in the *Methods* section, hydropower plants in northern British Columbia are excluded from the monthly generation estimates due to the limited spatial coverage of the applied meteorological forcing data. Although the headwater regions in the northern Fraser River Basin are omitted in the hydrologic simulations, hydropower facilities in the same river basin are located on different tributaries and are not directly influenced by the omitted headwater flowing into the mainstem. Furthermore, hydropower generation is estimated by scaling streamflow volumes, which can offset the missing watershed representation in the hydrologic simulations.

### Regulated streamflow

Once the mosartwmpy model routes the generated runoff through river networks and regulated reservoir storages, the streamflow outputs are validated with gauge observations (Environment Canada: Water Level and Flow, https://wateroffice.ec.gc.ca/) near hydropower plants. The observation stations are identified mostly at the downstream of hydropower plants, with additional upstream locations included when available. The streamflow evaluations calculate the modified KGE metric in Eq. (1) between the simulated and observed streamflow, including both natural and regulated conditions.

**Fig. 6:**
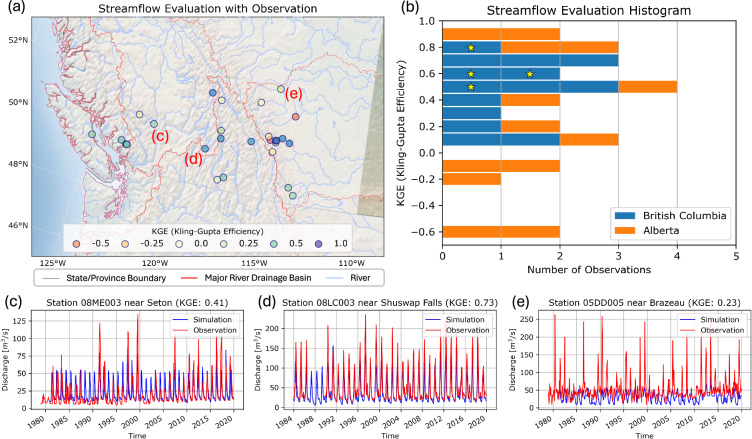
Evaluations of simulated streamflow against gauge observations near hydropower plants: (a) spatial distribution map, (**b**) histogram, and (**c**–**e**) example timeseries comparisons between simulations and observations of regulated streamflow. In panel (**b**), the evaluations using gauge observations of natural streamflow are indicated by yellow star markers, while the remaining evaluations represent those for regulated streamflow.

Figure 6 shows the KGE distributions, followed by example streamflow timeseries at selected locations. As shown in Fig. 6b, the overall statistics indicate a reasonable level of accuracy (about half of KGEs > 0.5) despite the limited model representation of reservoir operations (see *Streamflow Routing with Reservoir Operations* in the *Methods* section), except for certain observation locations in Alberta. The evaluations near run-of-river facilities with natural streamflow exhibit higher KGE values (yellow star markers), while those near facilities influenced by reservoirs show a relatively broad distribution of KGE values due to water management (no markers). One contributing factor to the low KGE values involves challenges in reproducing streamflow peaks during the high flow seasons, as shown in Fig. 6c–e. However, these discrepancies in streamflow peaks may be less critical for estimating hydropower generation as excessive streamflow cannot be fully exploited due to the mechanical constraints of hydropower plants. The deviations are apparent for observation locations near facilities along cascading reservoirs, which are commonly found across Western Canada. The KGE values show notable variation in the Canadian Rocky Mountains (around 115 °W and 48 °N in Fig. 6a). In these regions, certain streamflow observations directly measure water diversions by hydropower plants, which are not accurately represented by the river networks established in the mosartwmpy model.

### Estimated hydropower generation

The development of hydropower generation estimates in Western Canada aims to address the lack of publicly available monthly and utility-level datasets essential for power grid system studies. Given the limited availability of observations for validation, we present comparisons between the developed estimates and other accessible estimates or aggregated observations, which are listed in Table 1, to enhance the understanding of our newly developed dataset.

**Fig. 7:**
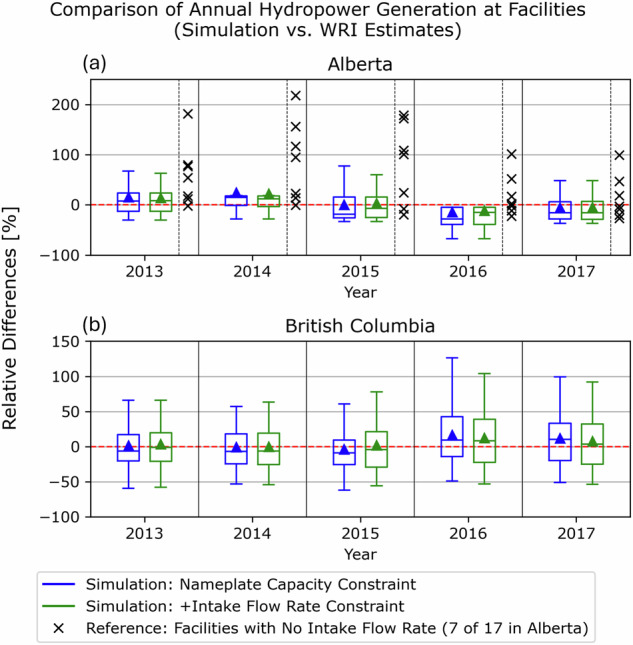
Comparisons of relative differences in annual hydropower generation with the WRI estimates^11^: (**a**) Alberta and (**b**) British Columbia. The blue and green box plots represent the distribution of relative difference for 103 hydropower plants, constrained by nameplate capacities and by additional intake flow rates, respectively. The red dashed line at zero indicates no difference. For reference, in panel (**a**), the black cross markers indicate the relative differences for the other seven facilities that lack water license information, constrained only by their nameplate capacities.

We first compare the hydropower generation estimates with the Global Database of Power Plants maintained by the World Resources Institute (WRI)^11^, which provides annual electricity generation estimates for individual power plants from 2013 to 2017. The WRI estimates were derived using a machine learning technique, the Gradient Boosting Regression Trees model. The independent variables for training include the nameplate capacities^11^, average capacity factors by region (EIA, https://www.eia.gov/electricity/data/eia923/) or by country (IRENA)^70^, as well as ERA5 streamflow order and runoff^38^ accumulations in their drainage areas^71^. Figure 7 shows the relative differences in annual total generation compared to the WRI estimates for 110 hydropower plants in Alberta and British Columbia, as defined in Eq. (4). The streamflow-hydropower scaling, constrained by nameplate capacities (blue box) or additional intake flow rates (green box), is compared with the WRI estimates. Over the five years, the average relative differences (triangular markers in the box plots) remain within 10% of the WRI estimates in British Columbia (Fig. 7b). The additional constraint that accounts for intake flow rates results in the deviations being even smaller (closer to the red line indicating no difference). Similarly, in Alberta (Fig. 7a), incorporating intake flow rate constraints improves the relative differences for facilities with water license information. The relative differences are large for the seven facilities where only the nameplate capacities are applied as a constraint (black cross markers). These estimate gaps can potentially decrease with the addition of constraints on intake flow rates. However, it is important to note that the WRI dataset serves as reference estimates based on statistical regression using machine learning techniques, with features such as runoff and nameplate capacity (total feature score > 0.75)^71^. Therefore, it should not be regarded as an accuracy measure for the simulated hydropower generation. Specifically, the WRI dataset has limitations due to the lack of training datasets outside of the U.S. regions, where reported hydropower generation datasets are heavily relied upon^71^.

4 $$\text{Relative Difference}\,[ \% ]=\frac{\text{Simulated Hydropower Generation}-\text{WRI Estimates}}{\text{WRI Estimates}}\times 100$$

**Fig. 8:**
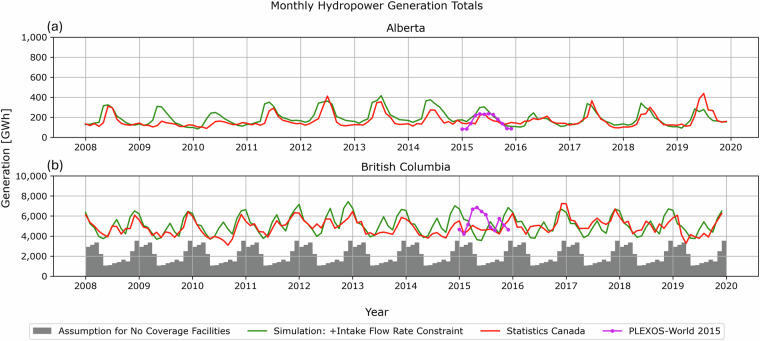
Comparisons of monthly hydropower generation totals with the Statistics Canada^3^ and PLEXOS-World 2015^9,10^ datasets: (**a**) Alberta and (**b**) British Columbia. The grey bars represent the generation totals of the hydropower plants excluded from the developed dataset, which are assumed to generate with the same monthly profiles obtained from WECC ADS 2032^34^.

The provincial statistics of monthly hydropower generation totals have been published by Statistics Canada^3^. To facilitate comparisons with the simulated hydropower generation, which has limited spatial coverage, we employ WECC ADS 2032^34^ for the facilities excluded in northern British Columbia (see Table 2), assuming that their monthly hydropower generation in 2008 repeat throughout the comparison periods. Figure 8 shows the simulated hydropower generation totals by province (green line) added on top of the assumed hydropower generation totals for the excluded facilities (grey bar). Although the assumed generation comprises a large portion during winter-peaking seasons, the combined estimates show comparable monthly magnitudes and seasonal variations in aggregated hydropower generation for both provinces.

For further comparison in Fig. 8, we additionally plot the PLEXOS-World 2015 dataset^9,10^ (purple line), which is aggregated for British Columbia and Alberta, respectively. In the dataset, monthly capacity factor profiles for individual hydropower plants were calibrated with the PLEXOS energy market model on hourly and global scales. For hydrologic modeling, streamflow downscaling^62^ was developed for runoff simulations using the Lund-Potsdam-Jena managed Land (LPJmL) model^72,73^, which were subsequently scaled for 2015 to match with the country-level mean capacity factors^70^ (Type 2, as classified by large-scale hydropower studies^36^). For British Columbia, our simulated hydropower generation totals suggest an inverse pattern of seasonal variations when compared to the PLEXOS-World 2015 dataset, aligning more closely with the Statistics Canada dataset. Notably, our dataset indicates that the monthly generation peaks of the PLEXOS-World 2015 dataset from April to July is less likely. The differences in seasonal generation patterns can be attributed to the water management modeling in streamflow simulations with informed constraints and hydropower scaling with the refined reference generation dataset, which demonstrates the advantages of the presented dataset. However, more extensive and detailed records of actual reservoir releases and hydropower generation are still necessary to improve their representation in integrated hydrology-hydropower modeling and to enable more robust technical validations.

## Usage Notes

The presented dataset provides monthly generation estimates for 110 hydropower facilities in Western Canada. A corresponding dataset for more than 1,400 facilities in the U.S., which is based on the same meteorological forcings, can be found in the following Zenodo repository^22,23^: https://zenodo.org/records/14269763. Consequently, both datasets can be implemented concurrently for transboundary power system studies across the WI region. Due to their differences in available observation records on flow and generation, the modeling approaches for reservoir operation rules and hydropower generation estimations are not identical even though they remain coincident. For adequate interpretation across the WI, it is highly recommended to check the referred dataset^23^ and its descriptor^22^ prior to using both datasets together.

The dataset aims to support robust, long-term power system planning under diverse water conditions. However, it should not be used to assess hydropower generation during extreme flood events when facilities may need to be disconnected from power grids due to dam safety and potential loss of control that could propagate into grid instability. Similarly, the dataset should not be utilized for unprecedented drought conditions where reservoir levels may fall below critical power pool levels. Furthermore, evolving water policies, including the Columbia River Treaty, can alter seasonal and monthly hydrological patterns. It is important to note that our hydropower generation dataset, which is derived based on Year 2008, does not account for any historical and future changes in environmental regulations, water management, or water policies.

## Data Availability

The VIC^26,27^ and mosartwmpy^31^ models are open-source and can be accessed with detailed descriptions at the following links: - VIC: https://vic.readthedocs.io/ - mosartwmpy: https://mosartwmpy.readthedocs.io/ The source codes used to generate the dataset are available in the GitHub repository: https://github.com/GODEEEP/tgw-hydro-canada.

## Appendix

**Table A-1:** A-1 List of Abbreviations.

Abbreviation	Definition
ADS	Anchor Data Set
CEM	Capacity Expansion Model
CONUS	Contiguous United States
DDS	Dynamically Dimensioned Search
EIA	Energy Information Administration
ERA	European Centre for Medium-Range Weather Forecasts Reanalysis
GRanD	Global Reservoir and Dams
GRFR	Global Reach-level Flood Reanalysis
HydroWIRES	Water Innovation for a Resilient Electricity System
IRENA	International Renewable Energy Agency
KGE	Kling-Gupta Efficiency
LPJmL	Lund-Potsdam-Jena managed Land
MODIS	Moderate Resolution Imaging Spectroradiometer
MOSART-WM mosartwmpy	Model for Scale Adaptive River Transport with Water Management
NACEI	North American Cooperation on Energy Information
NHN	National Hydrographic Network
OSTRICH	Optimization Software Toolkit for Research Involving Computational Heuristics
PCM	Production Cost Model
PNW	Pacific Northwest
U.S.	United States
WECC	Western Electricity Coordinating Council
WI	Western Interconnection
WRF	Weather Research and Forecasting
WRI	World Resource Institute
VIC	Variable Infiltration Capacity
